# Highly Luminescent and Anti-Photobleaching Core-Shell Structure of Mesoporous Silica and Phosphatidylcholine Modified Superparamagnetic Iron Oxide Nanoparticles

**DOI:** 10.3390/nano10071312

**Published:** 2020-07-04

**Authors:** Myeong Yun Kim, Jong-Pil Ahn, Seung Yun Han, Nam-Seob Lee, Young Gil Jeong, Do Kyung Kim

**Affiliations:** 1 Department of Anatomy, College of Medicine, Konyang University Hospital, Daejeon 35365, Korea; auddbs1357@naver.com (M.Y.K.); jjzzy@konyang.ac.kr (S.Y.H.); nslee@konyang.ac.kr (N.-S.L.); ygjeong@konyang.ac.kr (Y.G.J.); 2 Department of Business Cooperation Center, Korea Institute of Ceramic Engineering and Technology, Bucheon 14502, Korea; jpjam@kicet.re.kr

**Keywords:** europium complex, superparamagnetic, iron oxide, mesoporous silica, photoluminescent

## Abstract

Highly fluorescent magnetic nanoparticles (Eu(TTA)_3_(P(Oct)_3_)_3_@mSiO_2_@SPION) [europium (III) chloride hexahydrate = Eu; 4,4,4-trifluoro-1-(2-thienyl)-1,3-butanedione = TTA; trioctylphosphine = (P(Oct)_3_); mesoporous silica = mSiO_2_; superparamagnetic iron oxide nanoparticle = SPION] were developed as a dual-functional imaging agent. The hierarchical structure was composed of a magnetic core and mesoporous silica shell was constructed using a cationic surfactant template after coating with phosphatidylcholine of oleic acid coated SPION. Afterward, the surface and cavities of mSiO_2_@SPION were modified with 3-(trimethoxysilyl) propyl methacrylate (TMSPMA) as a silane coupling agent to introduce methacrylate groups. Eu(TTA)_3_(P(Oct)_3_)_3_ molecules are penetrated, located and bonded covalently inside of the cavities/mesopores of mSiO_2_, it shows extremely stable anti-photobleaching properties. The emission spectra of Eu(TTA)_3_(P(Oct)_3_)_3_@mSiO_2_@SPION indicated typical hypersensitivity transition ^5^D_0_→^7^F_2_ at 621 nm. The concentration of Eu(TTA)_3_(P(Oct)_3_)_3_@mSiO_2_@SPION was varied between 10 and 500 μL/mL to evaluate the cytotoxicity with NCI-H460 (H460) cells using MTT (3-(4,5-dimethylthiazol-2-yl)-2,5-diphenyltetrazolium bromide) assay. In addition, the presence of a strong red-emitting Eu(TTA)_3_(P(Oct)_3_)_3_@mSiO_2_@SPION in the cytoplasm was observed by fluorescence microscopy. Those results that it can be a potential candidate for dual-functional contrast agent and PL nanomaterials for fabricating the diagnostic kits to amplify the low signal.

## 1. Introduction

In recent years, nanomaterial has been attracted due to their versatile applications in biosensors [1], bio-imaging [2], drug delivery [3] and nanodevices [4], etc. In particular, in the area of bio-imaging field, imaging equipment and proper contrast agents are inevitable combination to overcome the detection limits at molecular and cellular levels at early stage of disease [5,6].

Bioimaging diagnostic equipment such as X-rays, magnetic resonance imaging (MRI), positron emission tomography (PET) and computer tomography (CT) are popular techniques in the medical field. In particular, MRI is known as one of the most widespread equipment that provides the functions of the non-invasive diagnostic application with a high spatial resolution and tomography, but has the disadvantage that it is low detection limit comparing to fluorescent bio-imaging tool [7]. The disadvantage of MRI can be resolved by using magnetic contrast agents such as gadolinium (T1 contrast agents) and superparamagnetic nanoparticles (T2 contrast agents) to enhance the signal response induced by external magnetic field.

Gadolinium-based contrast agents has been reported that those have limitations in obtaining high-resolution MRI images due to its short residence time in blood vessels and in vivo [8]. To replace gadolinium has been researched on MRI contrast agents based on SPION with relatively low toxicity and unique magnetic properties [9,10].

SPION has been synthesized by various methods such as microemulsions [11], coprecipitation [12], and thermal decomposition method [13]. The typical synthetic method of monodispersed SPION is known as the thermal decomposition of metallic oleate precursors [13]. This method is known to produce hydrophobic capped SPION with polar tail groups attached on surface. However, the hydrophobic properties of SPION limit the use in in vivo applications and dispersion in aqueous solutions. Therefore, oleic acid capped SPION was manipulated to alternate the surface properties by modifying their surface with hydrophilic moieties such as dextran [14], chitosan [15], polyethylene glycol [16] and silica [17,18].

In particular, mesoporous silica (mSiO_2_) has been widely used as a SPION surface coating material because of their chemical stability, convenient surface functionalization, biocompatibility, biodegradability and drug loading ability [19,20,21]. The magnetic core/mSiO_2_ shell nanoparticles coated with lanthanide (III) (Eu^3+^, Sm^3+^, Tb^3+^, and Dy^3+^) complexes have been actively developed as multifunctional bioimaging probes [22,23,24]. These magnetofluorescent nanoparticles can be feasible to use in vitro and in vivo applications. In this reason, those complicate structure have been intensively investigated to improve the disadvantages of conventional MRI contrast agents [25,26].

Among the lanthanide (III), Eu^3+^ complex emits very sharp red emission under UV light without photobleaching due to their effective intermolecular energy transfer from the coordinating ligand [27]. The photoluminescence (PL) properties of Eu^3+^ complex are strongly depending on constitutional composition of organic ligands. In addition, the carboxylic acid group of acrylic acid is strongly chelated with Eu^3+^ than the Eu^3+^ complex constituent molecules and protects the dissociation of Eu^3+^ complex under complicated physiological conditions [28]. Based on the above theory, a study was reported in which carboxylic acid activated polystyrene-based copolymer nanoparticles were prepared and then synthesized fluorescent nanoparticles through coordination bonding of carboxylic acid and chelated Eu^3+^ complex. As a result, those approaches proved that final composition has very high luminescent properties with an excellent anti-photobleaching [29]. However, although studies on fluorescent probes with core (magnetic)/shell(mSiO_2_) structures have been conducted so far, no studies on Eu(TTA)_3_(P(Oct)_3_)_3_ chelated on mSiO_2_@SPION have been reported.

In this work, we proposed multifunctional bioinert nanoparticles, i.e., SPION embedded mesoporous silica nanoparticles, based on mesopores modified by blocking nanopores with Eu(TTA)_3_(P(Oct)_3_)_3_, which work as luminescent moieties. In particular, phosphatidylcholine was introduced on oleic acid coated SPION. Hydrophobic ligand of phosphatidylcholine was attached on oleate moieties resulting in bilayer of liposome which is miscible in aqueous phase. The liposome layers on SPION have a strong affinity with silane group to enhance the homogeneous formation of mSiO_2_ on SPION.

The nanocavities of mSiO_2_ were used to protect β-diketone Eu^3+^ complex against photobleaching by covalent bonding the complex molecules inside of the cavities.

Multistep synthesis protocol was introduced to realize the complicated system. The synthesis procedure includes four steps: (I), SPION was synthesized by thermal decomposition; (II), After surface modification of SPION with phosphatidylcholine, nanoparticles having a magnetic core/silica shell structure were formed through a sol–gel process of TEOS using a cationic surfactant as an organic template; (III), Grafting the surface and cavities of mSiO_2_ with TMSPMA; (IV), Synthesizing Eu(TTA)_3_(P(Oct)_3_)_3_@mSiO_2_@SPION. The physical and chemical properties of the synthesized nanoparticles were evaluated, and it was investigated whether they have safe and strong photobleaching and high fluorescence properties in vivo.

## 2. Materials and Methods

### 2.1. Materials

Iron (III) chloride hexahydrate (FeCl_3_·6H_2_O), 3-(trimethoxysilyl) propyl methacrylate (TMSPMA, H_2_C=C(CH_3_)CO_2_(CH_2_)_3_Si(OCH_3_)_3_) and tetraethyl orthosilicate (TEOS, Si(OC_2_H_5_)_4_, 98%) were supplied by the Sigma-Aldrich Co., Ltd. (St. Louis, MO, U.S.A). Acrylic acid (AAc, CH_2_CHCOOH), sodium oleate (NaOl, CH_3_(CH_2_)_7_CHCH(CH_2_)_7_COONa), lecithin (phosphatidylcholine, soybean) and oleic acid (OA, CH_3_(CH_2_)_7_CHCH(CH_2_)_7_COOH) were obtained from Junsei Co., Ltd. (Tokyo, Japan). Ether (CH_3_CH_2_)_2_O, 99%), n-cetyltrimethylammonium bromide (CTAB, C_19_H_42_BrN), ethyl alcohol (EtOH, C_2_H_5_OH, 95%), chloroform (CHCl_3_, 99.8%) and n-hexane (CH_3_(CH_2_)_4_CH_3_, 96%) were purchased from Samchun Pure Chemical Co., Ltd. (Kyunggido, South Korea). Trioctylphosphine (P(Oct)_3_, C_24_H_51_P), RPMI-1640 medium (Hyclone, Waltham, MA, USA) and penicillin–streptomycin (Gibco, Waltham, MA, USA) were supplied by Thermo Fisher Scientific Co., Ltd. (Waltham, MA, USA). Ammonium nitrate (NH_4_NO_3_) was purchased from Daejung Chemicals Co., Ltd. (Siheung, Korea). Sodium hydroxide (NaOH) was obtained from Duksan pure chemicals Co., Ltd. (Kyunggido, South Korea). 1-octadecene (ODE, CH_3_(CH_2_)_15_CHCH_2_) was supplied by Wako Pure Chemical Industries Co., Ltd. (Osaka, Japan). Europium (III) chloride hexahydrate (Eu, EuCl_3_·6H_2_O) and 4,4,4-trifluoro-1-(2-thienyl)-1,3-butanedione (TTA, C_8_H_5_F_3_O_2_S) were supplied by Tokyo Chemical Industry Co., Ltd. (Tokyo, Japan). H460 cell was supplied by Korean Cell Line Bank (KCLB, Seoul, South Korea). Fetal bovine serum (FBS) was purchased from Welgene Biotech Co., Ltd. (Kyunggido, South Korea). The chemicals used in the experiment were without further purification process. Then, deionized water (DI water) used in the experiment was prepared by ELGA Flex 3 water purification system Midland VWS Co., Ltd. (High Wycombe, UK).

### 2.2. Characterization

The morphology and nanostructures of the synthesized mSiO_2_ were evaluated by JEM-ARM200 F field emission transmission electron microscopy (FE-TEM, 200 kV, JEOL) and JSM-7610 F field emission scanning electron microscope (FE-SEM, 10 kV, JEOL) with equipped energy dispersive X-ray (EDX) spectrometer. FT-IR spectra were recorded at the wavenumber ranges of 400–4000 cm^−1^ by Alpha FT-IR spectrometer equipped with platinum attenuated total reflection (Bruker, Billerica, MA, USA). The thermal behavior of prepared nanoparticles was measurements by TGA-50 and DSC-50 thermal analyzer (Shimadzu, Japan) and mSiO_2_ were heated from 50–600 °C with a heating rate of 10 °C/min in air. The specific surface area measurements of the sample were performed using Brunauer-Emmett-Teller (BET, Ga30093-2901, Micromeritics, MA, USA) N_2_ adsorption/desorption isotherm. Before physisorption measurements, the prepared mSiO_2_@SPION was degassed under vacuum at 400 °C for 4  h. Excitation and emission spectra of the samples were examined using a RF-5301PC (Shimadzu, Japan) photoluminescent quipped with a 150 W xenon lamp. The cytotoxicity of Eu(TTA)_3_(P(Oct)_3_)_3_@mSiO_2_@SPION was evaluated by MTT assay and then intercellular uptake of nanoparticles was confirmed using H460 cells. The presence of Eu(TTA)_3_(P(Oct)_3_)_3_@mSiO_2_@SPION in H460 cells was observed by Leica DM 2500 fluorescence microscope equipped with PE 300 cool LED.

### 2.3. Synthesis of Iron (III) Oleate (FeOl) Complex

**Scheme 1:**
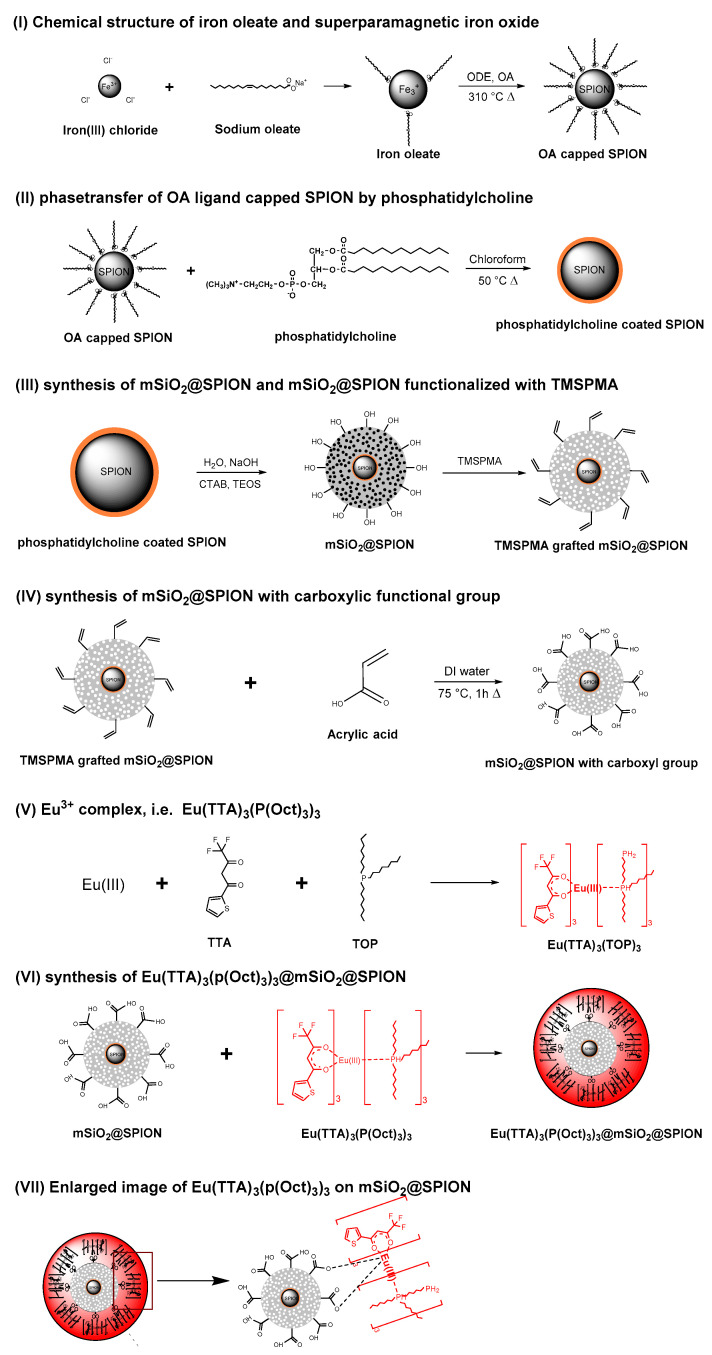
Schematic illustration of (**I**) thermal decomposition of monodispersed superparamagnetic iron-oxide nanoparticles (SPION), (**II**) phosphatidylcholine modification on SPION, (**III**) Methacrylate activation on mSiO_2_@SPION with TMSPMA, (**IV**) activation of carboxyl group on mSiO_2_@SPION, (**V**) formation of Eu^3+^ complex with TTA and P(Oct)_3_, (**VI**) Eu(TTA)_3_(P(Oct)_3_)_3_ chelated on mSiO_2_@SPION and enlarged image of Eu(TTA)_3_(P(Oct)_3_)_3_ on mSiO_2_@SPION. (VII) Enlargement of chemical structure of Eu(TTA)_3_(P(Oct)_3_)_3_ on mSiO_2_@SPION.

The preparation of FeOl was carried out with a slight modification of the previously reported method [13]. FeOl is produced by an ion exchange of iron (III) chloride hexahydrate and NaOl. The procedure for FeOl complex is illustrated in Scheme 1. In 500 mL beaker, iron chloride hexahydrate (10.8 g, 40 mmol) and NaOl (36.5 g, 120 mmol) were dissolved in 80 mL of EtOH, 60 mL of DI water and 140 mL of n-hexane. The 500 mL beaker containing the mixture was sealed and stirred at 70 °C for 3 h. When the reaction was complete, the resulting solution was kept in freezer for 5 h. The liquid at bottom layer was removed from the frozen organic layer and 80 mL of deionized (DI) water was added to wash the organic layer. The solution was frozen in freezer for 2 h and once again the upper layer containing FeOl was separated from the lower layer. The above performed process was repeated 3 times to collect the upper layer containing FeOl. The collected final FeOl solution was dried at room temperature overnight to evaporate the residual n-hexane. The solid waxy form of FeOl was stored in the freezer.

### 2.4. Production of Monodisperse Superparamagnetic Iron-Oxide Nanoparticles (SPION)

The OA ligand-capped SPION was synthesized by thermal decomposition of the metal precursor in an organic solvent [13]. The synthetic procedure of the SPION is described in Scheme 1 (I). A quantity of 3.6 g of the FeOl precursor synthesized in a 100 mL flask (two-necked round bottom) and 0.57 g of OA was dissolved in 20 g of ODE. After connecting the proportional integral derivative (PID) control module to heating mantle and a magnetic stirrer, 100 mL flask (two-necked round bottom) containing the mixture was placed in a heating mantle and stirred for 1 h at 110 °C to evaporate physical water in the mixture. Then the mixture containing FeOl precursor was heated to reflux at 310 °C and the temperature was maintained at 310 °C for 30 min and then the solution was cooled at room temperature. The synthesized SPION was washed several times using n-hexane, EtOH and acetone (1:2:1, V/V/V), and the solution was centrifuged at 21,500× *g* for 20 min. The OA-capped SPION was redispersed in chloroform and stored in 50 mL vial (0.6 g/mL).

### 2.5. Hydrophobic to Hydrophilic Phase Transfer of OA Ligand Capped SPION by Phosphatidylcholine

The hydrophobic oleic acid ligand capped SPION was phase-transferred by the addition of liposome-based phosphatidylcholine. The process of capping phosphatidylcholine on the surface of SPION was as follows. Briefly, 0.5 mL of SPION dispersed in chloroform was placed into a 2 mL EP tube, respectively. Then, 1 mL ether and 0.5 mL EtOH were added into EP tubes. The 2 mL EP tube containing SPION was sonicated for 3 min. Afterward, the sample was precipitated by centrifuge at 21,500× *g* for 5 min. After precipitation, the supernatant was remover and 0.5 mL of chloroform and 80 mg of phosphatidylcholine were added to EP tube containing precipitated SPION. The mixture was sonicated at 50 °C for 1 h, then 0.5 mL of ether and 1 mL of EtOH were added into EP tube and centrifuged at 21,500× *g* for 10 min. The SPION coated phosphatidylcholine were redispersed in 2 mL of chloroform. The overall procedure for phosphatidylcholine coated SPION was briefly described in Scheme 1 (II).

### 2.6. Synthesis of mSiO_2_@SPION by Sol–Gel Process

The mSiO_2_@SPION was synthesized by slightly modifying the method reported by J. L. Nyalosaso et al. [30] 240 mL of 45.73 mM CTAB aqueous solution was placed in a 500 mL two necked round bottom flask and then 1.76 mL of 2-M NaOH was added. The mixture solution was sonicated at 70 °C for 1 h. After sonication, 2 mL of phosphatidylcholine coated SPION dispersed in chloroform was very slowly added. The mixture solution was sonicated for an additional 1 h at 70 °C to evaporate the residual chloroform. The flask immersed in water bath was placed on a magnetic stirrer and stirred under reflux with a stirring speed of 750 rpm at 40 °C for 1 h. To construct a mesoporous silica layer on the surface of SPION, 400 μL TEOS was injected very slowly and kept for 30 min. Afterward, 800 μL TEOS was additionally added at a rate of 1 drop per 5 sec and reacted for 2 h. After 2 h later, the temperature was increased to 80 °C and reacted for 2 h. The reaction flask was cooled to room temperature while stirring. The CTAB was extracted by dispersing the sample in NH_4_NO_3_ EtOH solution (10 mg/mL) at 80 °C for 24 h. The sample was washing three times with 200 mL EtOH and centrifuging. Subsequently, the precipitated mSiO_2_@SPION was redisposed in 20 mL of EtOH, placed in a 50-mL tube and stored. The overall reaction procedure of mSiO_2_@SPION was shown in Scheme 1 (III).

### 2.7. Activation of Carboxylate Group on mSiO_2_@SPION

The anchorage of silanol group on mSiO_2_ was introduced with TMSPMA. Briefly, mSiO_2_@SPION dispersed in 20 mL ethanol with a concentration of 0.6 g/mL was transferred in 50 mL tube, 100 μL TMSPMA and 0.6 mL dilute acetic acid (1:10 glacial acetic acid: DI water) were added and sonicated for 5 min. The sample was transferred to 2 mL EP tube and then centrifuged at 21,500× *g* for 5 min. After precipitation, the supernatant was decantated and redisposed in EtOH and centrifuged again. The washing procedure was repeated 3 times. The obtained silane modified mSiO_2_ was dispersed in 100 mL of EtOH (6 g/L, ammonium nitrate/EtOH) in 250 mL flask (two-necked round bottom), then flask was sonicated at 80 °C for 3 h to eliminate CTAB from the nanopores in mSiO_2_. After 3 h, the mixture was washed with 200 mL × 3 EtOH and precipitated by centrifuge. The sample was redispersed in 30 mL DI water and stored in 50-mL tube. The activation of carboxylic group on the surface of TMSPMA modified mSiO_2_@SPION was formed by free radical polymerization with AAc. Thirty milliliters of TMSPMA grafted mSiO_2_@SPION dispersed in DI water transferred into 100-mL flask (two-necked round bottom) and then additionally 27 mL of DI water, 3 mL of PPS (1.75 g of PPS dissolved in 50 mL of DI water) and 0.6 mL of AAc were added. The reaction flask was sonicated at 75 °C for 1 h. The impurities and unreacted chemicals were dialyzed using a dialysis bag (Mw. Cutoff = 12,400) against 5 L of DI water. The dialyzed DI water was exchanged with fresh 5 L DI water 5 times every 2 h. The reaction scheme for carboxyl group activation on mSiO_2_@SPION was shown in Scheme 1 (IV).

### 2.8. Preparation of β-Diketone Europium Complex (Eu(TTA)_3_(P(Oct)_3_)_3_)

The Eu(TTA)_3_(P(Oct)_3_)_3_ was prepared according to the previously reported method [29]. The chemical structure of Eu(TTA)_3_(P(Oct)_3_)_3_ was described in Scheme 1 (V) One micromole (0.366 g) Eu^3+^ was dissolved in 50 mL of EtOH. TTA stock solution was prepared by dissolving 1 mmol (0.222 g) TTA in 50 mL EtOH. P(Oct)_3_ stock solution was also prepared by dissolving 1 mmol (0.37 g) to 50 mL EtOH. The concentrations of stock solution were as follows; Eu^3+^: 20 mM, TTA: 20 mM, P(Oct)_3_:20 mM. Ten milliliters of DI water and 100 μL, 20 mM of Eu^3+^ stock solution and 300 μL, 20 mM of TTA and 300 μL, 20 mM of P(Oct)_3_ were added to 20 mL glass vial, respectively. Then 20 μL of concentrated ammonia solution was added. The 20 mL glass vial containing mixture was stirred with magnetic stirrer in water bath at 60 °C to 600 rpm for 2 h. After stirring, the resulting β-diketone Eu^3+^ complex was stored at room temperature.

### 2.9. Synthesis of Eu(TTA)_3_(P(Oct)_3_)_3_ Chelated Eu(TTA)_3_P(Oct)_3_)_3_@mSiO_2_@SPION

The chemical structure of the β-diketone europium complex was chelated with a carboxylic acid ligand on mSiO_2_@SPION is shown in Scheme 1 (VI). The synthesis of Eu(TTA)_3_(P(Oct)_3_)_3_@mSiO_2_@SPION was carried out in a 100-mL flask (two-necked round bottom), in which 50 mL of mSiO_2_@SPION was dispersed in DI water. Then, 1 mL Eu(TTA)_3_(TOP)_3_ into 50 mL of mSiO_2_@SPION dispersed in 100-mL flask (two-necked round bottom). Then 0.5 μL of concentrated ammonia solution was injected to the flask containing the mixture. The 100 mL flask containing mixture was stirred in a water bath at 70 °C to 700 rpm for 1 h. The formed Eu(TTA)_3_(P(Oct)_3_)_3_@mSiO_2_@SPION are dialyzed against 5 L DI water using a dialysis bag (Mw. Cutoff = 12,400). DI water was changed 5 times every hour and left overnight.

### 2.10. Cell Culture

The H460 cells with epithelial morphology were cultured in RPMI-1640 medium added with FBS (10%) and penicillin-streptomycin (1%). The H460 cells were grown in 100 mm size cell dishes. The H460 cells were passaged when approximately 80% confluence was reached. The H460 cells were washed twice with PBS. PBS in the culture plate was completely removed and then 1 mL trypsin was added (3 min at 37 °C). Then, 2 mL RPMI-1640 medium was added to collect a solution containing the H460 cells and transferred into a 15 mL tube. The tube was centrifuged at 1300 rpm for 3 min to obtain cell pellets. After centrifugation, the medium was removed from tube, and 3 mL of fresh RPMI- 1640 medium was added, and the cell pellet is resuspended. Subsequently, suspended H460 cells were dispensed into three different 100 mm culture dishes, respectively. The medium used for cell culture was replaced with a fresh medium every 3 days. H460 cells were cultured in incubator under human conditions.

### 2.11. Eu(TTA)_3_(P(Oct)_3_)_3_@mSiO_2_@SPION Uptake

The H460 cells were cultured onto each coverslip (diameter = 18 mm) with 1 × 10^5^ cells/coverslip in 24 well culture dishes for 24 h. Then reseeded H460 cells were treated with Eu(TTA)_3_(P(Oct)_3_)_3_@mSiO_2_@SPION dispersed in culture medium for 24 h incubation and washed twice with PBS. The H460 cells treated with Eu(TTA)_3_(P(Oct)_3_)_3_@mSiO_2_@SPION were fixed with 4% paraformaldehyde (PFA) solution for 15 min at 4 °C. After washing the sample with PBS, the H460 cells were DAPI-stained and observed under fluorescence microscopy. All fluorescence microscope magnifications were obtained at 400× magnification.

### 2.12. Cytotoxicity Assay

MTT (3-(4,5-dimethylthiazolyl-2)-2,5-diphenyltetrazolium bromide) assay was performed to evaluate the toxicity of the prepared Eu(TTA)_3_(P(Oct)_3_)_3_@mSiO_2_@SPION. Briefly, H460 cells were seeded in 96-well plates at a density of 1 × 10^5^ cells/well and incubated at 37 °C in 5% CO_2_ incubator for 24 h before the addition of Eu(TTA)_3_(P(Oct)_3_)_3_@mSiO_2_@SPION. Seven different concentrations (10 μg/mL, 50 μg/mL, 100 μg/mL, 200 μg/mL, 300 μg/mL and 500 μg/mL Eu(TTA)_3_(P(Oct)_3_)_3_@mSiO_2_@SPION) were treated to H460 cells and incubated for 24 h without exchanging the media. After incubation, 10 μL MTT solution (5 mg/mL) is added and then cells were further incubated for 4 h. The formazan product was dissolved in 100 µL dimethyl sulfoxide (DMSO). The optical density of the H460 cells was then measured using a microplate reader. In addition, the absorbance values of cells treated at each concentration were expressed in percentages.

## 3. Results and Discussion

Scheme 1 illustrates overall synthetic procedures of Eu(TTA)_3_(P(Oct)_3_)_3_@mSiO_2_@SPION. The FeOl is produced by an ion-exchange of Fe^3+^ and Na^+^ from sodium oleate (NaOl). In typical, octadecene with high boiling point (315 °C) is used as a non-coordinating solvent to synthesize OA-capped SPION by thermal decomposition of metallic complex. The SPION capped with hydrophobic oleic chain is easily dispersed in a nonpolar solvent. However, oleic acid layer on SPION repels silane molecules during the synthesis of mSiO_2_@SPION resulting in separation of mSiO_2_ and SPION instead of core-shell structure. To overcome this problem, phosphatidylcholine is introduced on oleic acid coated SPION. The phosphatidylcholine is composed of choline head group and glycerophosphate and is known to contain various fatty acids (fatty acid and unsaturated fatty acid). The primary micelle structure is formed by van der Waals force action between the alkyl tail present in phosphatidylcholine and the hydrophobic alkyl tail of OA present on the surface of the magnetic core. Mesoporous silica shell layer is formed using a typical sol-gel reaction based on an organic template (CTAB). Methacrylate group with TMSPMA as a coupling silane agent is capable of free radical polymerization on mSiO_2_@SPION. Finally, β-diketone Eu^3+^ complex is chelated with a carboxylic acid ligand on mSiO_2_@SPION.

**Figure 1:**
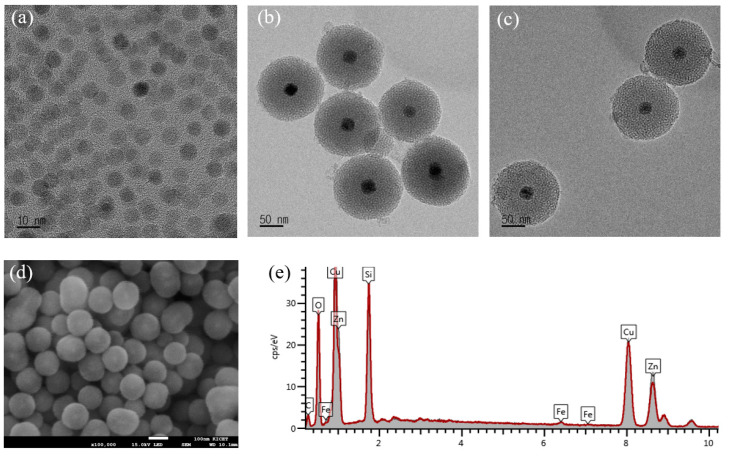
TEM images for morphologic structure analysis of synthesized nanoparticles. (**a**) OA-capped SPION, (**b**) mSiO_2_@SPION, (**c**) mSiO_2_@SPION after modifying with β-diketone Eu^3+^ complex (**d**) SEM image of mSiO_2_@SPION and (**e**) EDX analysis results of mSiO_2_@SPION.

The morphologies of mSiO_2_@SPION with magnetic (core)/silica (shell) structure are characterized by FE-TEM and FE-SEM images. The OA-capped SPION formed by thermal decomposition of FeOl precursors is shown in Figure 1a. The image shows that magnetic nanospheres with an average size of about 12 nm were successfully synthesized. The phosphatidylcholine and hydrophobic tail of OA on SPION form a primary micelle work as a nucleation site for mesoporous silica shell with an average size of 120 nm as shown in Figure 1b. An average pore size measured with TEM results is 30 Å with a standard deviation of 0.47.

Figure 1c shows mSiO_2_@SPION after modifying with β-diketone Eu^3+^ complex. According to the TEM image, a distinctive morphologic difference is not monitored after the modification of Eu(TTA)_3_(P(Oct)_3_)_3_. In addition, the shape of mSiO_2_@SPION was confirmed once again by SEM (Figure 1d). The SEM image shows that mSiO_2_@SPION has a spherical structure with the magnetic core as bright spots. The EDX analysis results are shown in Figure 1e. The peaks of zinc and copper are coming from the sample holder.

**Figure 2:**
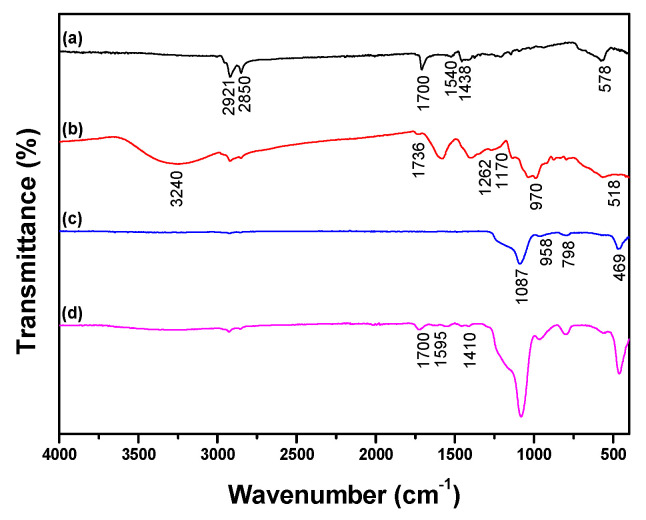
FT-IR spectra of nanospheres synthesized for the formation of fluorescent magnetic nanoparticles with a magnetic core/silica shell structure. (**a**) oleic acid (OA)-capped SPION, (**b**) SPION modified with phosphatidylcholine, (**c**) mSiO_2_@SPION and (**d**) Eu(TTA)_3_(P(Oct)_3_)_3_@mSiO_2_@SPION.

The surface properties of the nanoparticles in each modification steps are evaluated by FT-IR spectrophotometer. The peak at 578 cm^−1^ in Figure 2a FT-IR spectrum attributes to the presence of Fe-O bonding from Fe_3_O_4_ [31,32]. The peak at 1438 cm^−1^ is corresponding to the symmetric vibration of the iron carboxylate on SPION. Also, the peak at 1540 cm^−1^ is representing the COO^-^ and the peak at 1700 cm^−1^ indicates the presence of C=O stretching of the COO^-^ [31,33]. The peaks at 2921 cm^−1^ and 2850 cm^−1^ show C-H stretching peaks from OA. Figure 2b shows FT-IR spectrum of SPION modified with phosphatidylcholine and several new transmittance peaks are observed. The broad peak at about 3240 cm^−1^ shown in Figure 2b is corresponds to OH group presented on mSiO_2_@SPION. The peak at around 1736 cm^−1^ is corresponding to C=O stretching of the ester group indicating the presence of phosphatidylcholine (phosphatidyl ethanol amine and phosphatidyl serine) and the peak at 1262 cm^−1^ corresponds to a P-O-R bonds. The peak at 1170 cm^−1^ corresponds to a C-O (stretch ester), and the peak at 970 cm^−1^ shows the presence of choline containing phospholipids. Additionally, the small pike at 518 cm^−1^ is represented phosphate group (O-P-O). As show in Figure 2c, the peaks appeared at 1087 cm^−1^, 789 cm^−1^ and 470 cm^−1^ of mSiO_2_@SPION spectrum indicate the presence of Si-O-Si (stretching vibration) bond. In addition, the peaks appeared at 3442 cm^−1^, and 958 cm^−1^ are attributed to stretching vibration of Si-OH (silanol group) and free silanol groups due to hydrogen bonding to the nanoparticles, respectively. Figure 2d shows the FT-IR spectrum of Eu(TTA)_3_(P(Oct)_3_)_3_@mSiO_2_@SPION. The peaks at 1595 cm^−1^ and 1410 cm^−1^ indicate Eu^3+^ chelated with carboxylic acid of AAc formed by free radical polymerization of TMSPMA presented on mSiO_2_@SPION and the peak at 1700 cm^−1^ is corresponding to C=O stretch. These results indicate that surface modifications, shell layer formation and chelation of Eu^3+^ complex for Eu(TTA)_3_(P(Oct)_3_)_3_@mSiO_2_@SPION at each stage are successfully conducted.

**Figure 3:**
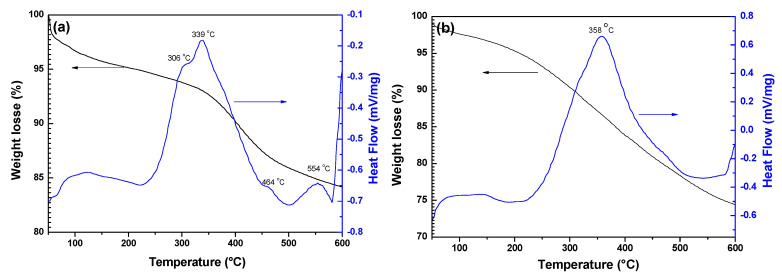
DSC-TGA profiles of (**a**) mSiO_2_@SPION and (**b**) Eu(TTA)_3_(P(Oct)_3_)_3_@mSiO_2_@SPION.

The characteristic thermal behaviors of mSiO_2_@SPION and Eu(TTA)_3_(P(Oct)_3_)_3_@mSiO_2_@SPION are measured by TGA and DSC (Figure 3). The TGA curves in Figure 3a, b show a rapid weight loss at 130 °C region from room temperature because they correspond to the dehydration adsorbed on the particles. The secondary weight loss appears in the range of 300 °C to 500 °C corresponds to oxidation of -CH_3_ and evaporation of residual organic solvent. The weight loss of mSiO_2_@SPION is much lower than that of Eu(TTA)_3_(P(Oct)_3_)_3_ chelated mSiO_2_@SPION, demonstrating the presence of chelated Eu^3+^ complex on mSiO_2_@SPION. There is little weight loss around 600 °C, indicating the presence of organic molecules such as TEOS are remained in the synthesized nanoparticles. The weight loss is calculated to be mSiO_2_@SPION: 17.42% and Eu complex@mSiO_2_@SPION: 27.38%, respectively. As observed in TGA curves, the endothermic peak of DSC observed at 130 °C corresponds to the dehydration of absorbed water. The exothermic peak in a temperate range of 250–500 °C is related to the decomposition of organic molecules. It can be assumed that the heat of reaction increased due to the presence of the chelated Eu^3+^ complex on mSiO_2_@SPION.

**Figure 4:**
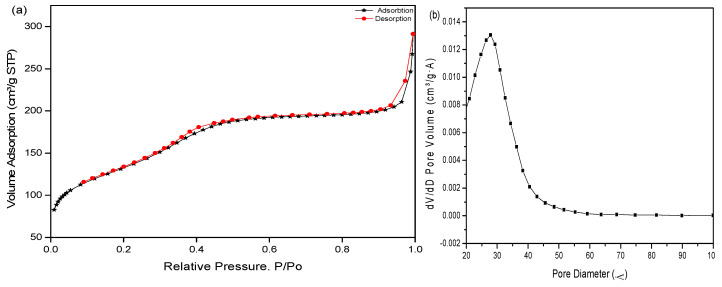
(**a**) Nitrogen adsorption–desorption isotherm plot of mSiO_2_@SPION and (**b**) pore size of mSiO_2_@SPION synthesized with a core/shell structure to be homogenized.

The BET and average pore diameter (d_p_) are measured using adsorption isotherms in the relative pressure (P/P^o^) range of 0.05 to 0.3 according to the previously reported method [34]. mSiO_2_@SPION is degassed at 400 °C for four hours before N_2_ adsorption evaluation. In Figure 4a the nitrogen physisorption isotherm of mSiO_2_@SPION shows an IV isotherm with a strong absorption trend around P/P^o^ = 0.9–1.0, indicating the existence of typical mesoporous structures. The pore size and distribution curve of mSiO_2_@SPION are shown in Figure 4b. Based on BET model calculation, mSiO_2_@SPION has an average pore size of about 28 Å with a specific surface area of 464.96 m^2^/g.

**Figure 5:**
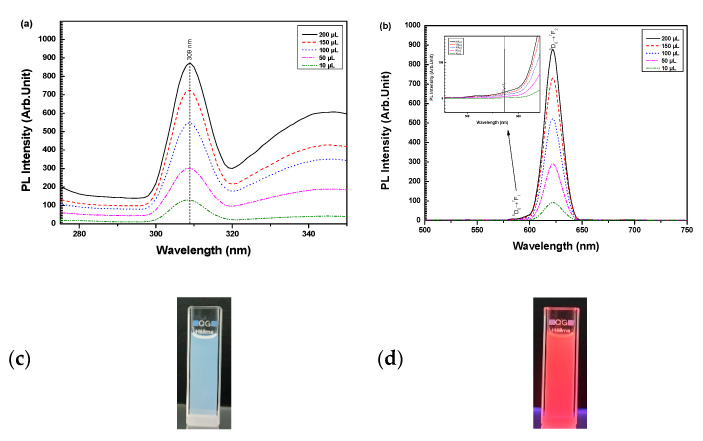
(**a**) Excitation (309 nm) and (**b**) emission (621 nm) spectra depending on different concentrations of Eu(TTA)_3_(P(Oct)_3_)_3_@mSiO_2_@SPION. Photographic images of Eu(TTA)_3_(P(Oct)_3_)_3_@mSiO_2_@SPION diluted in DI water at (**c**) room light and (**d**) UV light.

Figure 5 shows photoluminescence (PL) characteristics of (a) excitation and (b) emission spectra depending on different concentrations of Eu(TTA)_3_(P(Oct)_3_)_3_@mSiO_2_@SPION. The inherent f-f transition peak of the Eu^3+^ in Eu(TTA)_3_(P(Oct)_3_)_3_@mSiO_2_@SPION is evaluated by the PL measurements. The excitation spectrum of Eu(TTA)_3_(P(Oct)_3_)_3_@mSiO_2_@SPION shows the typical optical tendency due to f-f transition of Eu^3+^ complex with strong red emission characteristics [35]. Figure 5b the strong emission peak at 621 nm is due to ^5^D_0_→^7^F_2_ transition. The peak with lower intensity appeared at 592 nm can be assigned to ^5^D_0_→^7^F_1_ transition. The main peaks appeared at 592 nm with small shoulder and 621 nm with main pike correspond to the transitions from ^5^D_0_ state and can be allocated to ^5^D_0_→^7^F_1_ (592 nm) and ^5^D_0_→^7^F_2_ (621 nm). Based on the PL results, the PL properties are not drastically influenced after introducing the Eu^3+^ complex into mSiO_2_@SPION. The intensity ratio (^5^D_0_→^7^F_2_/^5^D_0_→^7^F_1_) of Eu(TTA)_3_(P(Oct)_3_)_3_@mSiO_2_@SPION define that Eu(TTA)_3_(P(Oct)_3_)_3_@mSiO_2_@SPION has a strong PL properties especially in ^5^D_0_→^7^F_2_ transition coming from stable Eu^3+^ complex. The excitation spectrum (λ_em_ = 621 nm) of the Eu(TTA)_3_(P(Oct)_3_)_3_@mSiO_2_@SPION is identified a maximum peak at 309 nm. As the concentration of Eu(TTA)_3_(P(Oct)_3_)_3_@mSiO_2_@SPION is gradually increased, the PL intensity of the major ^5^D_0_→^7^ F_2_ at 621 nm is linearly enhanced. As a result, Eu(TTA)_3_(P(Oct)_3_)_3_ will improve the PL intensity with a strong red emission in the UV range. Figure 5c,d shows photographic images of Eu(TTA)_3_(P(Oct)_3_)_3_@mSiO_2_@SPION diluted in DI water under room light and UV light.

**Figure 6:**
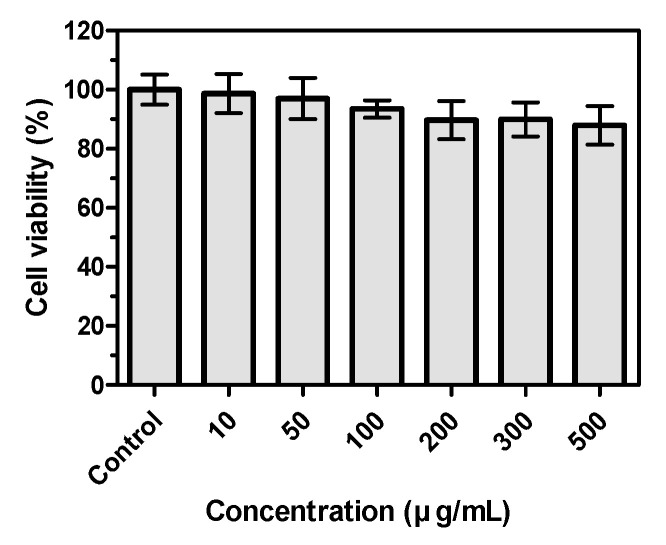
Cytotoxicity evaluation of Eu(TTA)_3_(P(Oct)_3_)_3_@mSiO_2_@SPION in H460 cells are incubated for 24 h with different concentrations (10, 100, 300 and 500-μg/mL Eu(TTA)_3_(P(Oct)_3_)_3_@mSiO_2_@SPION) and their viability is evaluated by MTT assay against untreated control cells. Error bars indicate mean ±SD (n = 12).

The cytotoxicity of as-prepared Eu(TTA)_3_(P(Oct)_3_)_3_@mSiO_2_@SPION on H460 cells and PBS as a control group are performed at seven different concentrations (10, 50, 100, 200, 300 and 500 μg/mL Eu(TTA)_3_(P(Oct)_3_)_3_@mSiO_2_@SPION). Eu(TTA)_3_(P(Oct)_3_)_3_@mSiO_2_@SPION induced a dose-dependent decline in cell viability. The obtained results suggest that all treated samples do not influence any cytotoxic effect to H460 cells after 24 h incubation. As shown in Figure 6, all treated cases reveal at least 90% H460 cell viability. Nevertheless, extremely lower cytotoxicity at 10 and 50 μg/mL are shown, which can be assigned to inert biocompatibility of mSiO_2_, as well as hydrophobic properties of Eu^3+^ complex. Moreover, all the chemical moieties used to manipulate these hierarchical structures are widely well studied in nanomedicinal applications. In this reason, the suggested composition of Eu(TTA)_3_(P(Oct)_3_)_3_@mSiO_2_@SPION are biocompatible without inducing any cytotoxicity. However, some cytotoxicity appeared in the experiment are inevitable due to the colloidal stability of dispersion of Eu(TTA)_3_(P(Oct)_3_)_3_@mSiO_2_@SPION. Eventually, the particles will be sedimented at the bottom of the culture plate and it will be cover on the adherent H460 cells resulting in several negative effects, i.e., oxidative stress, generation of reactive oxygen species (ROS), interruption of physiological function, will be happened in cellular level. Eventually, the coverage of the particles on cellular surface will induce the mechanical stress resulting in interruption of intercellular signal and nutrition uptake into the cells. As a result, apoptosis and autophagy will be induced in most case of nanoparticle uptake.

**Figure 7:**
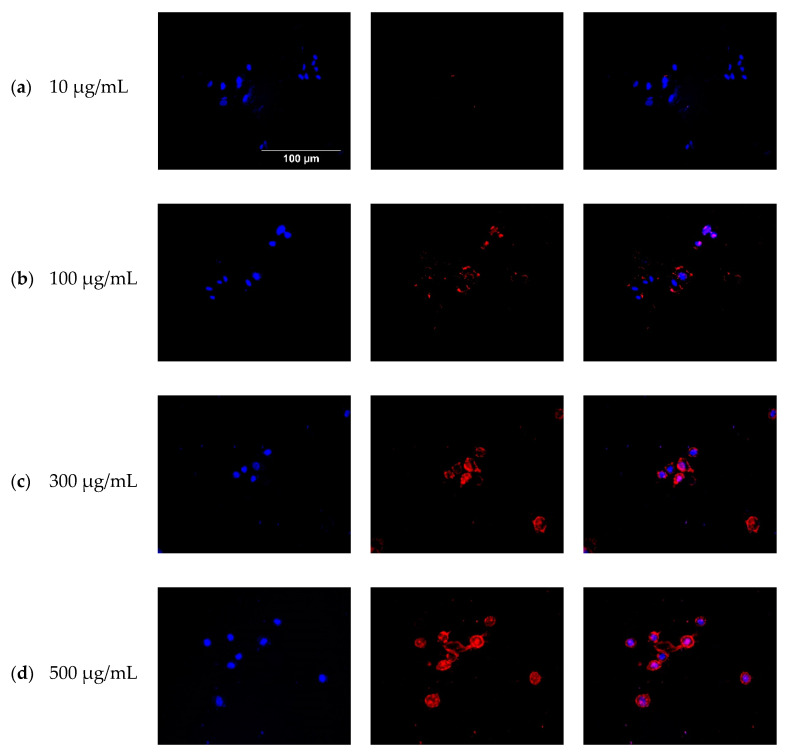
Intercellular uptake images of H460 cells treated with different concentrations: the concentration of the Eu(TTA)_3_(P(Oct)_3_)_3_@mSiO_2_@SPION of (**a**) 10 μg/mL, (**b**) 100 μg/mL, (**c**) 300 μg/mL and (**d**) 500 μg/mL.

The intercellular uptake and localization of mSiO_2_@SPION functionalized with fluorescent moieties of Eu(TTA)_3_(P(Oct)_3_)_3_ into H460 cells is evaluated using fluorescence microscope. The H460 cells are treated with four different concentrations (10 μg/mL, 100 μg/mL, 300 μg/mL and 500 μg/mL Eu(TTA)_3_(P(Oct)_3_)_3_@mSiO_2_@SPION) after 24 h incubation. The H460 cells treated with Eu(TTA)_3_(P(Oct)_3_)_3_@mSiO_2_@SPION are stained with DAPI. To demonstrate the cellular uptake of Eu(TTA)_3_(P(Oct)_3_)_3_@mSiO_2_@SPION, the quantity of internalized nanoparticles is assessed by population and intensity of red fluorescence in H460 cells. By increasing the dose concentration, the fluorescence intensity is proportionally enhanced. Figure 7 shows intercellular uptake images of H460 cells treated with (a) 10 μg/mL, (b) 100 μg/mL, (c) 300 μg/mL and (d) 500 μg/mL Eu(TTA)_3_(P(Oct)_3_)_3_@mSiO_2_@SPION. The images confirmed Eu(TTA)_3_(P(Oct)_3_)_3_@mSiO_2_@SPION intracellular localization and also that fluorescent intensities increased when the number of added Eu(TTA)_3_(P(Oct)_3_)_3_@mSiO_2_@SPION per cell were increased. In the concentration of 10 μg/mL, the uptake of Eu(TTA)_3_(P(Oct)_3_)_3_@mSiO_2_@SPION is ignorable due to the detection limit of instrument. When the concentration of Eu(TTA)_3_(P(Oct)_3_)_3_@mSiO_2_@SPION was increased to 100 μg/mL, red emitting part begin to appear due to endocytosis. Based on the results, the optimum concentration of Eu(TTA)_3_(P(Oct)_3_)_3_@mSiO_2_@SPION to visualize the cellular structure is around 500 μg/mL.

Intercellular uptake of nanoparticles is strongly correlated with their size and surface properties, i.e., cell preferred ligands and hydrophobicity, etc. H460 cell population of cellular fluorescence increased from 0 to 24 h and continually increased up to 72 h treatment. In general, nanoparticles conjugated with organic fluorescent dyes will be endocytosed within first few hour of treatment, intercellular accumulation of nanoparticles will be saturated up to 24 h and fluorescent properties are gradually decrease due to photobleaching, intercellular degradation of organic molecules and/or exocytosis. However, Eu(TTA)_3_(P(Oct)_3_)_3_@mSiO_2_@SPION suggested in this work shows extremely stable against photobleaching and biocompatibility. Eu(TTA)_3_(P(Oct)_3_)_3_ molecules are penetrated, located and bonded covalently inside of the cavities/mesopores of mSiO_2_, it shows rather stable anti-photobleaching. Eu(TTA)_3_(P(Oct)_3_)_3_ existed inside of the mSiO_2_ cavities avoids straight interaction with physiological environments resulting in excluding the intercellular degradation of the molecules. In particular, the hydrophobic properties of Eu(TTA)_3_(P(Oct)_3_)_3_ on the surface of mSiO_2_@SPION will drastically enhance the endocytosis because it has more strong affinity with cell membranes because of their hydrophobic characteristics.

## 4. Conclusions

In this study, we demonstrated that the multifunctional bioinert nanoparticles, i.e., SPION embedded mesoporous silica nanoparticles-based on mesoporous capillaries modified by blocking nanopores with Eu(TTA)_3_(P(Oct)_3_)_3_ work as luminescent moieties. In particular, immiscible-layer OA-capped SPION against TEOS was changed to miscible layer with phosphatidylcholine. Therefore, the core-shell structure of mSiO_2_@SPION were successfully synthesized by forming the micelles on the surface of SPION.

In general, nanopore of mSiO_2_ was used as a cargo to encapsulate the hydrophobic drugs and release the therapeutic molecules in physiological environments. However, we used opposite concept to protect β-diketone Eu^3+^ complex against photobleaching by covalent bonding the complex molecules inside of the cavities. In this way, external light sources, i.e., UV and laser, are indirectly exposed to protect β-diketone Eu^3+^ complex when those PL particles are used to visualize the biologic samples. It will prolong the lifetime of fluorescent properties for several months together with encapsulated Eu^3+^ complex will decrease the cytotoxicity because β-diketone Eu^3+^ complex will not be located inside of cavities without reaching out.

These results imply that, due to their low cytotoxicity, good biocompatibility, highly PL properties with red-emitting color and magnetic nanoparticle embed structure, Eu(TTA)_3_(P(Oct)_3_)_3_@mSiO_2_@SPION are potential candidate for dual-functional contrast agent and PL nanomaterials for fabricating the diagnostic kits to amplify the low signal.
